# Supplementary material for: Resource-aware construct design in mammalian cells
Source: Nat Commun. 2023 Jun 16;14:3576. doi: 10.1038/s41467-023-39252-4 (PMC10275982; doi:10.1038/s41467-023-39252-4)
Supplement: Supplementary file 1 — Supplementary information [file 41467_2023_39252_MOESM1_ESM.pdf]

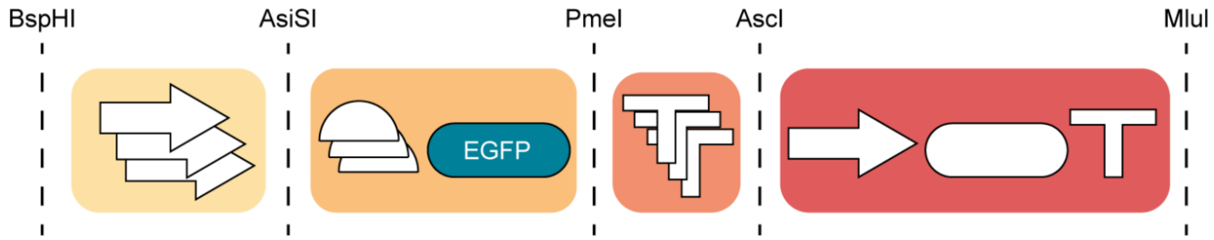

Fig. S1. Modular plasmid architecture adopted in this study. A modular plasmid was designed to easily substitute genetic parts of interest for mammalian gene expression. The plasmid is composed of a promoter driving the expression of an EGFP where the BspHI and AsiSI restriction sites flank the promoter, AsiSI and PmeI flank the Kozak-EGFP cassette, and PmeI and AscI flank the polyA. Thus, individual elements can be replaced by digestion with combinations of restriction enzymes. The plasmid also allows for easy insertion of a second TU using AscI and MluI.

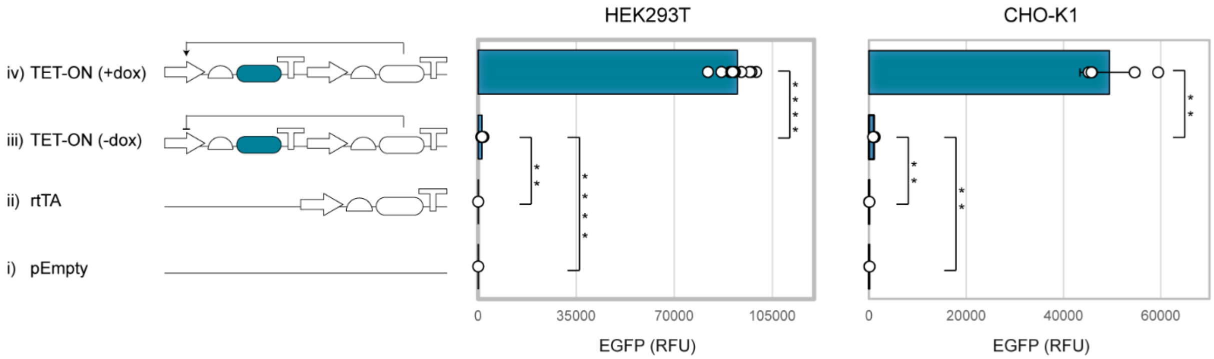

Fig. S2. Test plasmid output from synthetic constructs of increasing complexity. Synthetic designs with increasing complexity are transfected in HEK293T (left) and CHO-K1 (right) cells. An empty plasmid (i), a construct constitutively expressing the rtTA transactivator (ii), and the TET-ON inducible system without (iii) and with (iv) dox addition (1 ng/µl) were considered. Test plasmid output is reported as mean RFU ± std. Number of biological repeats for each sample is reported in Supplementary data file 3. Two sided Mann-Whitney test *P* value: ****<0.0001, ***<0.0005, **<0.005, *<0.05. Exact P values can be found in Supplementary data file 4. Data analysis is described in the Methods section and Supplementary Note 1. Source data are provided in the Source Data file.

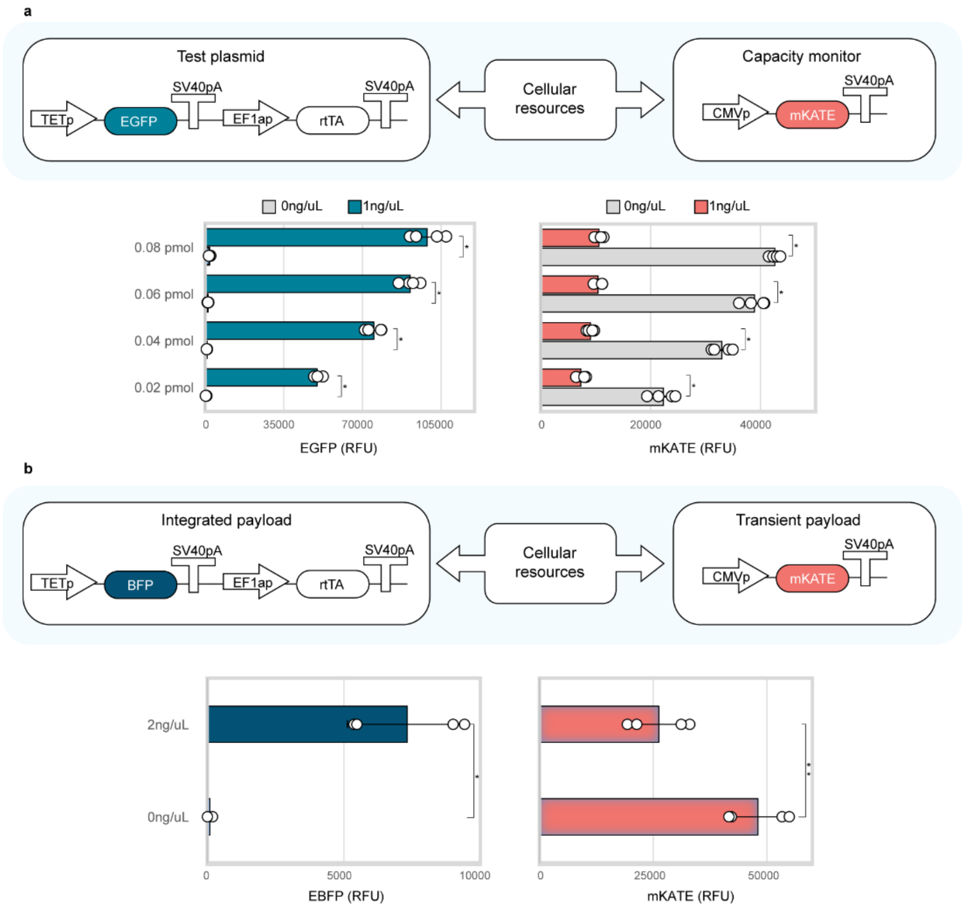

Fig. S3. Decreasing gene dosage does not rescue resource competition. (a) Resource competition between co-expressed genes when different test and monitor plasmids dosages are co-transfected in HEK293T. **(b)** Resource competition between a single-copy integrated (dox-inducible BFP) and a transient payload (constitutive mKATE) in HEK293T. Test plasmid and capacity monitor expression levels are reported as mean RFU ± std. Number of biological repeats for each sample is reported in Supplementary data file 3. Two sided Mann-Whitney test P value: ****<0.0001, ***<0.0005, **<0.005, *<0.05. Exact P values can be found in Supplementary data file 4. Data were analysed as described in the Methods section and Supplementary Note 1. Source data are provided in the Source Data file.

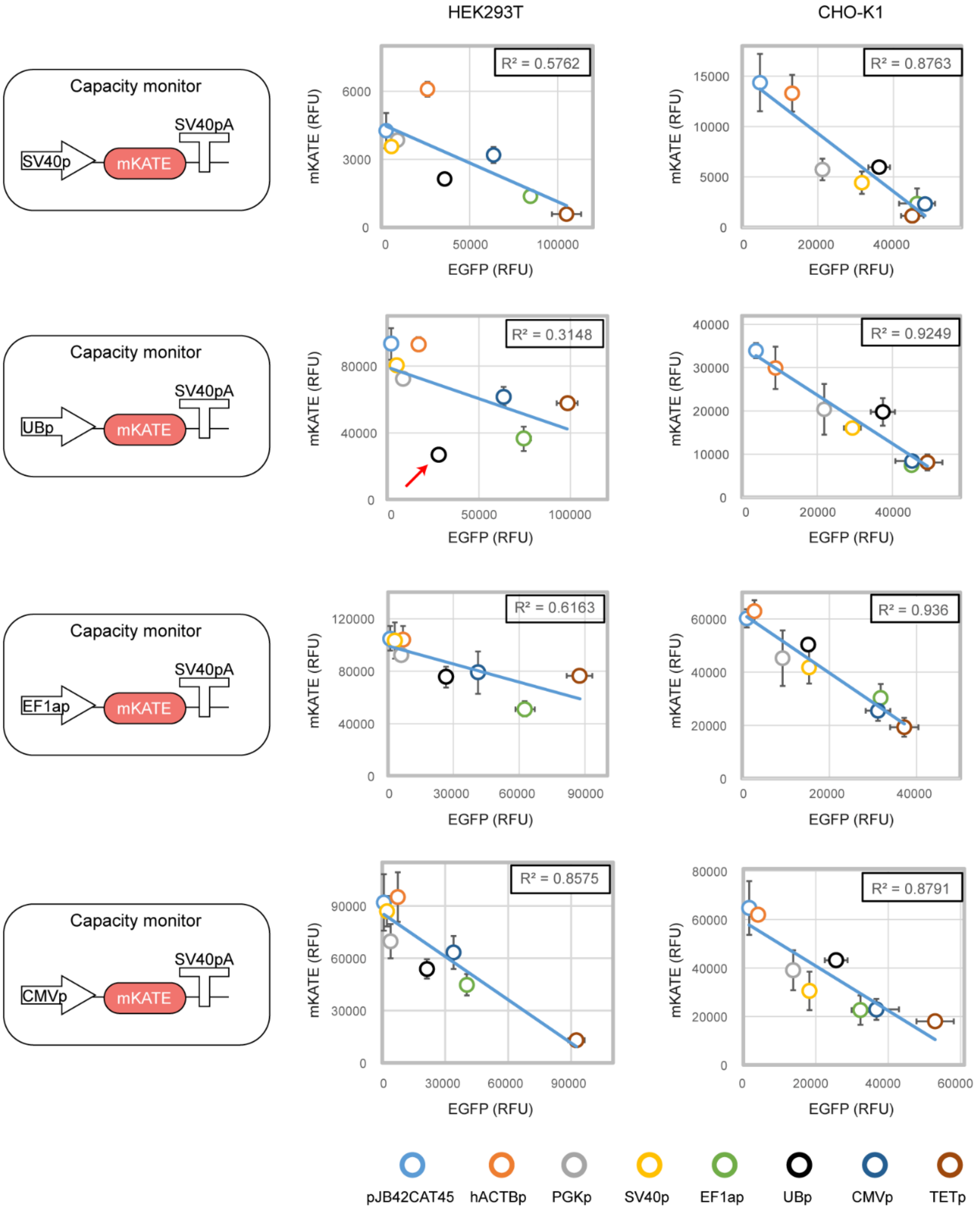

Fig. S4. Response of different capacity monitor designs to resource load. Resource load imposed by seven constitutive promoters and one inducible promoter was measured with four different capacity monitor designs in HEK293T (left panels) and CHO-K1 (right panels). The red arrow indicates competition between UBp-mKATE (capacity monitor) and UBp-EGFP (test plasmid). Test plasmid and capacity monitor expression levels are reported as mean RFU ± std. Number of biological repeats for each sample is reported in Supplementary data file 3. Data were analysed as described in the Methods section and Supplementary Note 1. Source data are provided in the Source Data file.

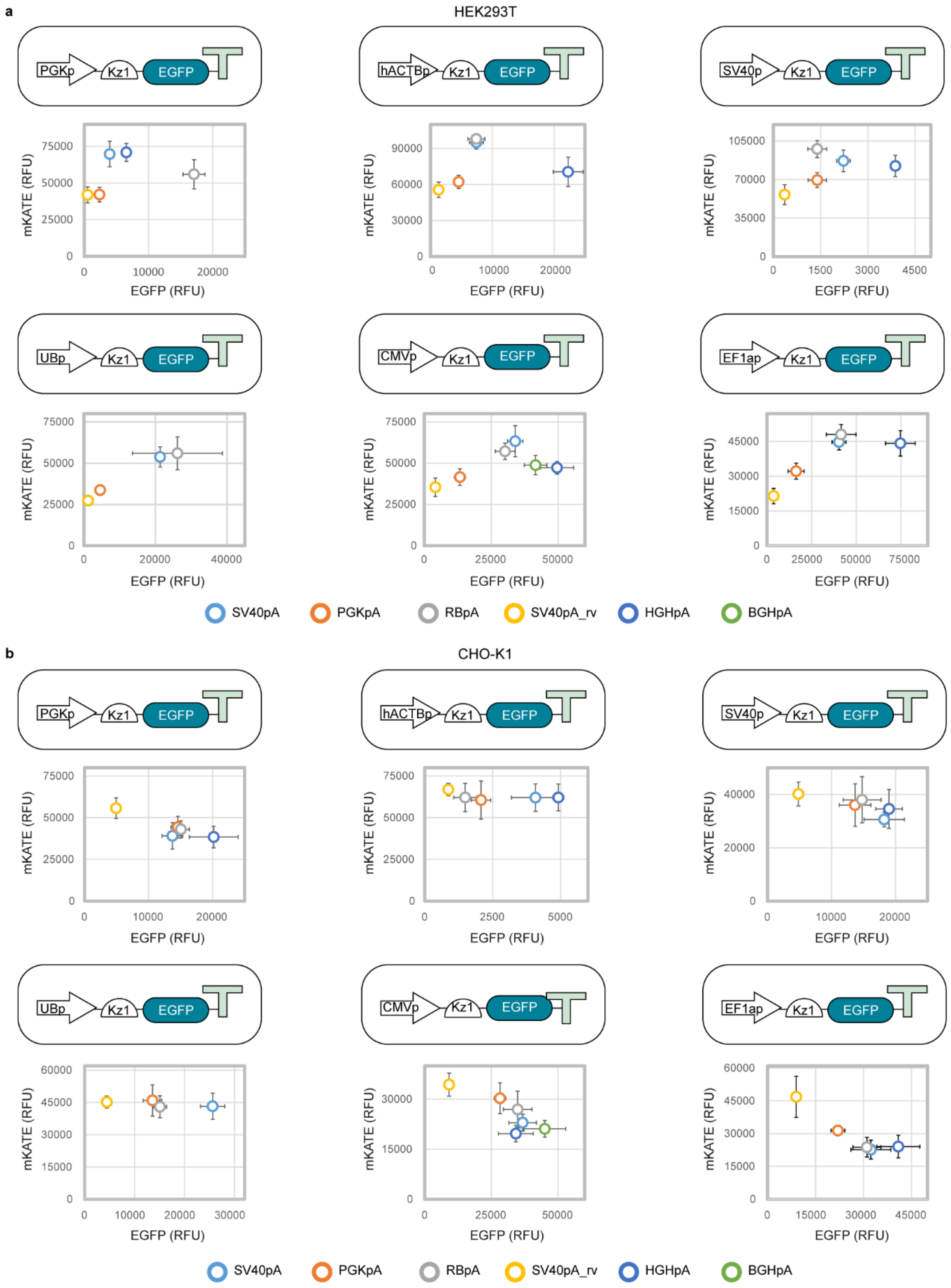

Fig. S5. Resource competition imposed by different polyA sequences. Impact of different promoter-polyA configurations on the capacity monitor expression in **(a)** HEK293T and **(b)** CHO-K1 cells. Test plasmid and capacity monitor expression levels are reported as mean RFU ± std. Number of biological repeats for each sample is reported in Supplementary data file 3. Data were analysed as described in the Methods section and Supplementary Note 1. Source data are provided in the Source Data file.

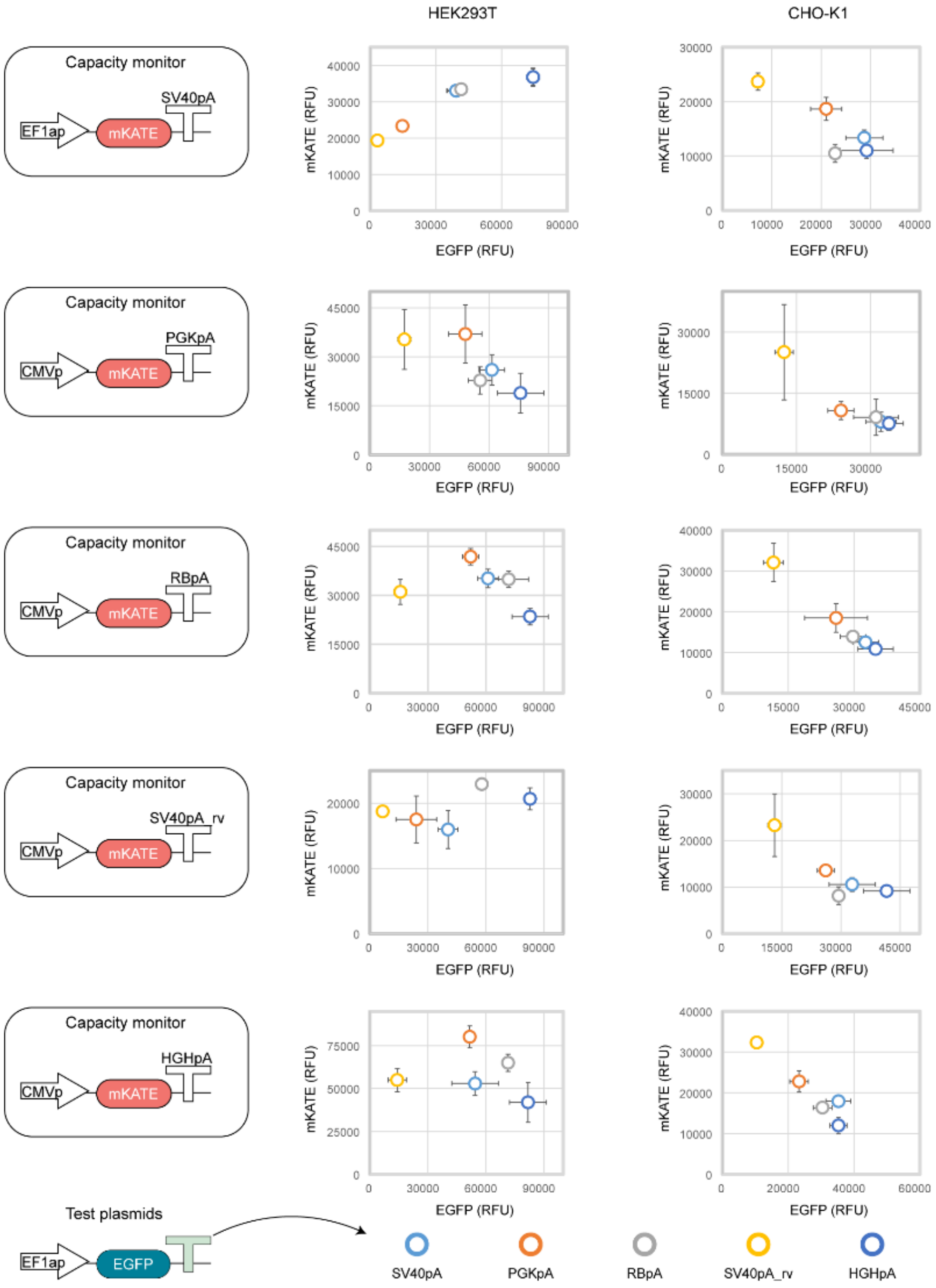

Fig. S6. Sequence- and cell line-dependent polyA-based resource competition. The resource load of test constructs bearing five polyAs downstream of EF1ap-Kz1-EGFP was measured in HEK293T (left panels) and CHO-K1 (right panels). The constructs were tested in competition with five different capacity monitors engineered to contain one of five polyA sequences. Test plasmid and capacity monitor expression levels are reported as mean RFU ± std. Number of biological repeats for each sample is reported in Supplementary data file 3. Data were analysed as described in the Methods section and Supplementary Note 1. Source data are provided in the Source Data file.

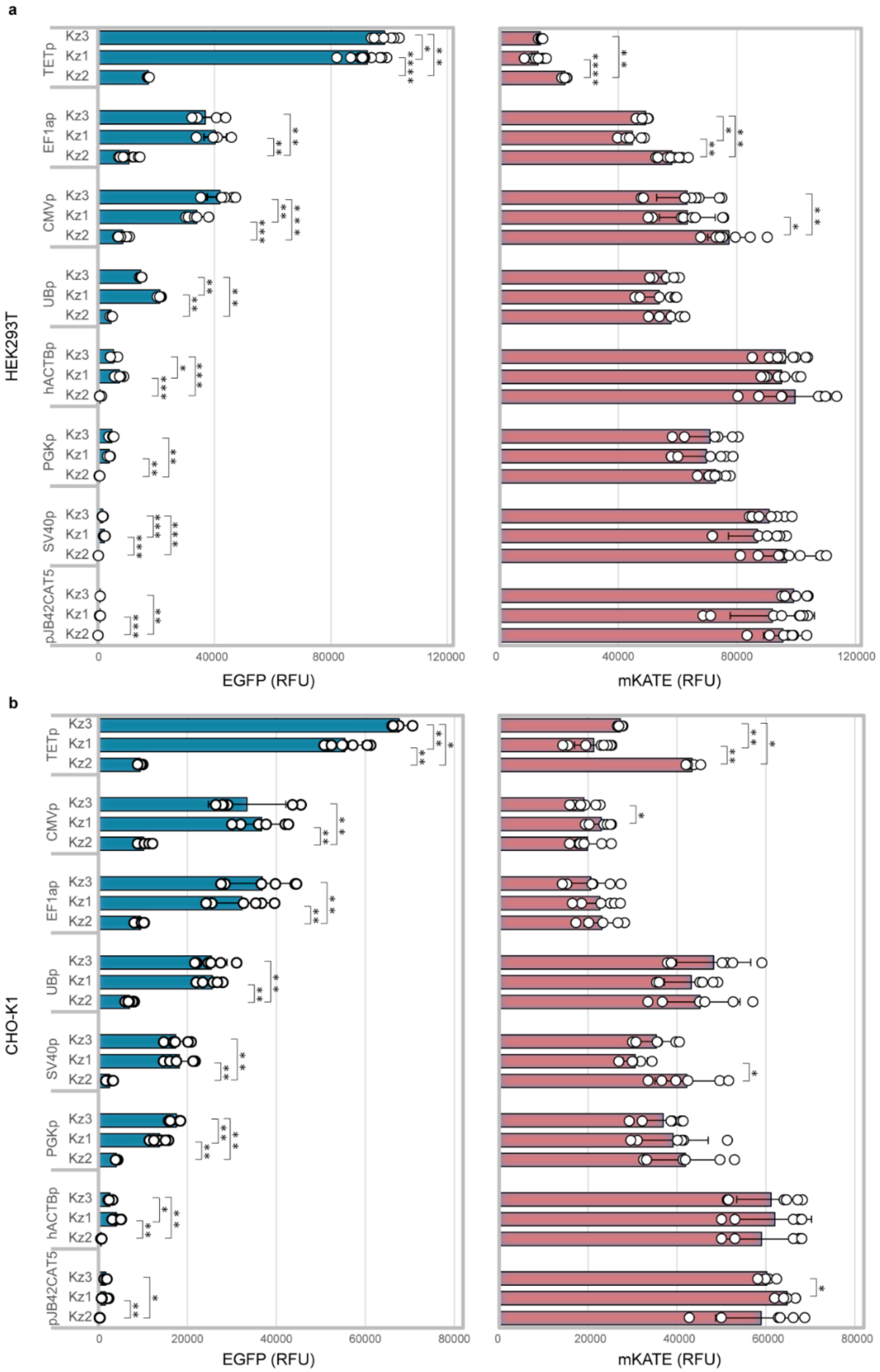

Fig. S7. Resource competition imposed by different Kozak sequences. Impact of different promoter-Kozak configurations on the expression of the capacity monitor in **(a)** HEK293T and **(b)** CHO-K1 cells. Test plasmid and capacity monitor expression levels are reported as mean RFU ± std. Number of biological repeats for each sample is reported in Supplementary data file 3. Two sided Mann-Whitney test *P* value: ****<0.0001, ***<0.0005, **<0.005, *<0.05. Exact P values can be found in Supplementary data file 4. Data were analysed as described in the Methods section and Supplementary Note 1. Source data are provided in the Source Data file.

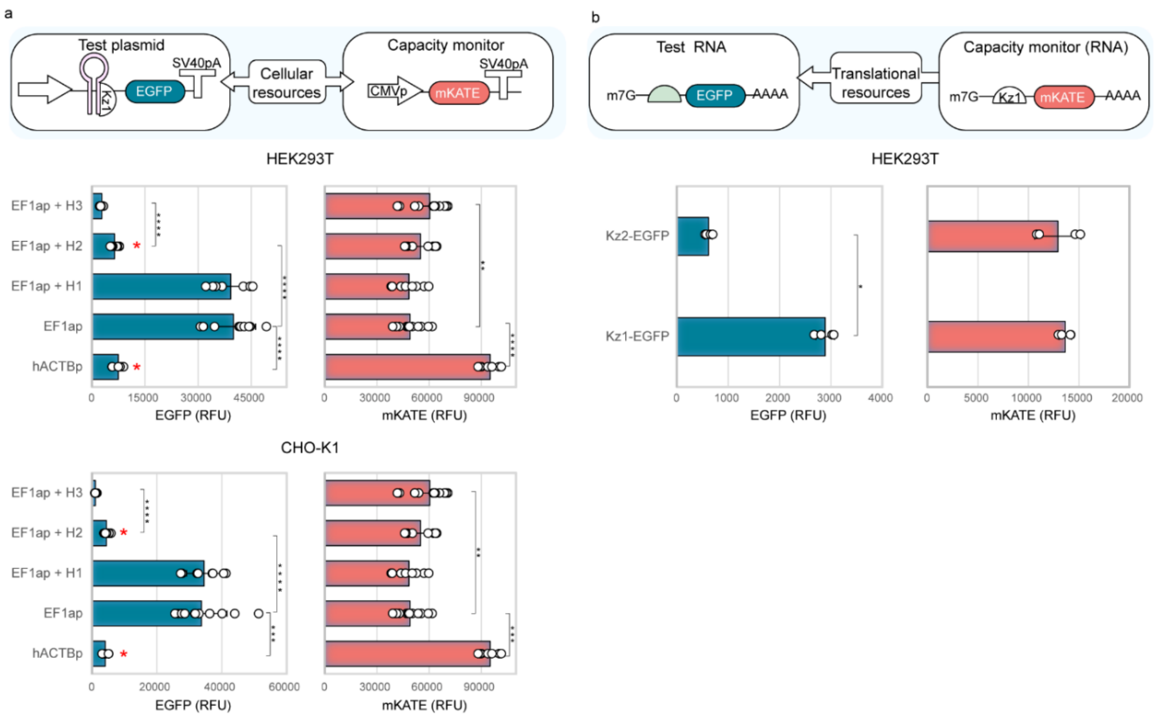

Fig. S8. Impact of translational efficiency on resource competition profiles. (a) Three hairpins (H1, H2 and H3) were cloned at the 5`-UTR of the EF1ap-Kz1-EGFP-SV40pA construct. Each hairpin displays a different efficiency in sequestering the Kozak sequence, thus modulating translation. The impact of the designs on the capacity monitor was compared with that of two designs with no hairpin bearing either EF1ap or hACTBp in HEK293T (top panel) and CHO-K1 (bottom panel). Red asterisks identify two constructs with similar EGFP output, one with a stronger promoter and H2, and one with a weaker promoter and no hairpin. **(b)** Two test mRNAs bearing Kozak sequences with different strength (Kz1-EGFP and Kz2-EGFP) and one monitor mRNA (Kz1-mKATE) were generated by *in-vitro* transcription. All mRNAs were capped and polyadenylated. Test mRNAs and monitor mRNA were co-transfected in HEK293T. Expression levels are reported as mean RFU ± std. Number of biological repeats for each sample is reported in Supplementary data file 3. Two sided Mann-Whitney test P value: ****<0.0001, ***<0.0005, **<0.005, *<0.05. Exact P values can be found in Supplementary data file 4. Data were analysed as described in the Methods section and Supplementary Note 1. Source data are provided in the Source Data file.

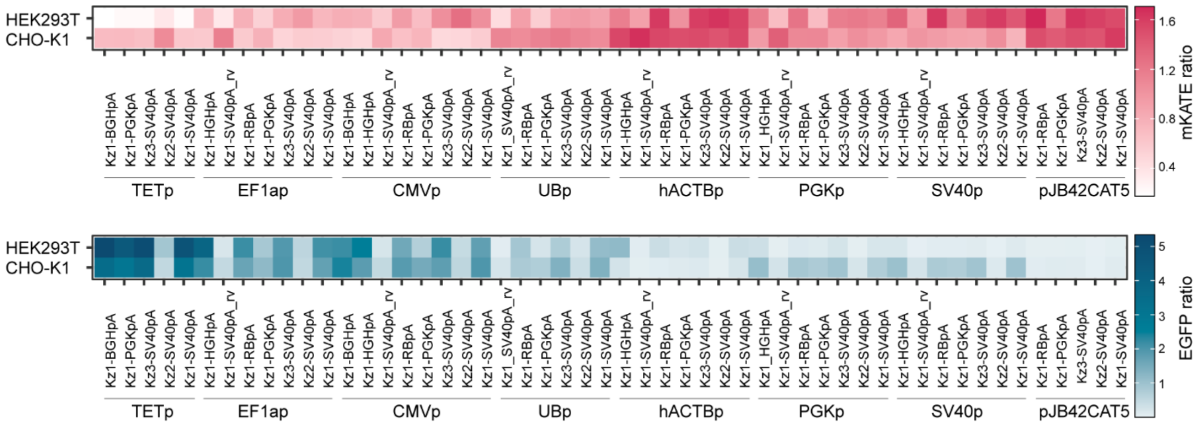

Fig. S9. Resource competition imposed by the complete library. Test plasmid and capacity monitor expression levels for the entire library. Test plasmid and capacity monitor expression levels are reported as mean RFU values normalised to the average RFU of the complete library. Number of biological repeats for each sample is reported in Supplementary data file 3. Data were analysed as described in the Methods section and Supplementary Note 1. Source data are provided in the Source Data file.

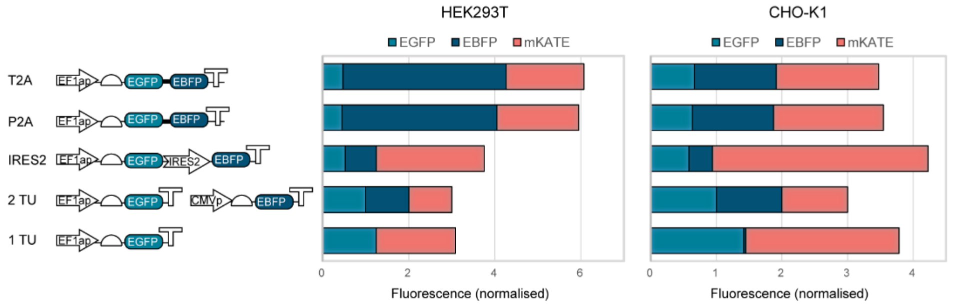

Fig. S10. Test plasmid and capacity monitor outputs of multi-gene constructs. Test plasmid (EGFP, EGFP) and capacity monitor (mKATE) expression for single and multi-gene designs. EGFP, EBFP, and mKATE expression levels are reported as mean RFU values normalised to the fluorescence value of the corresponding reporter in the 2TU configuration. Number of biological repeats for each sample is reported in Supplementary data file 3. Data were analysed as described in the Methods section and Supplementary Note 1. Source data are provided in the Source Data file.

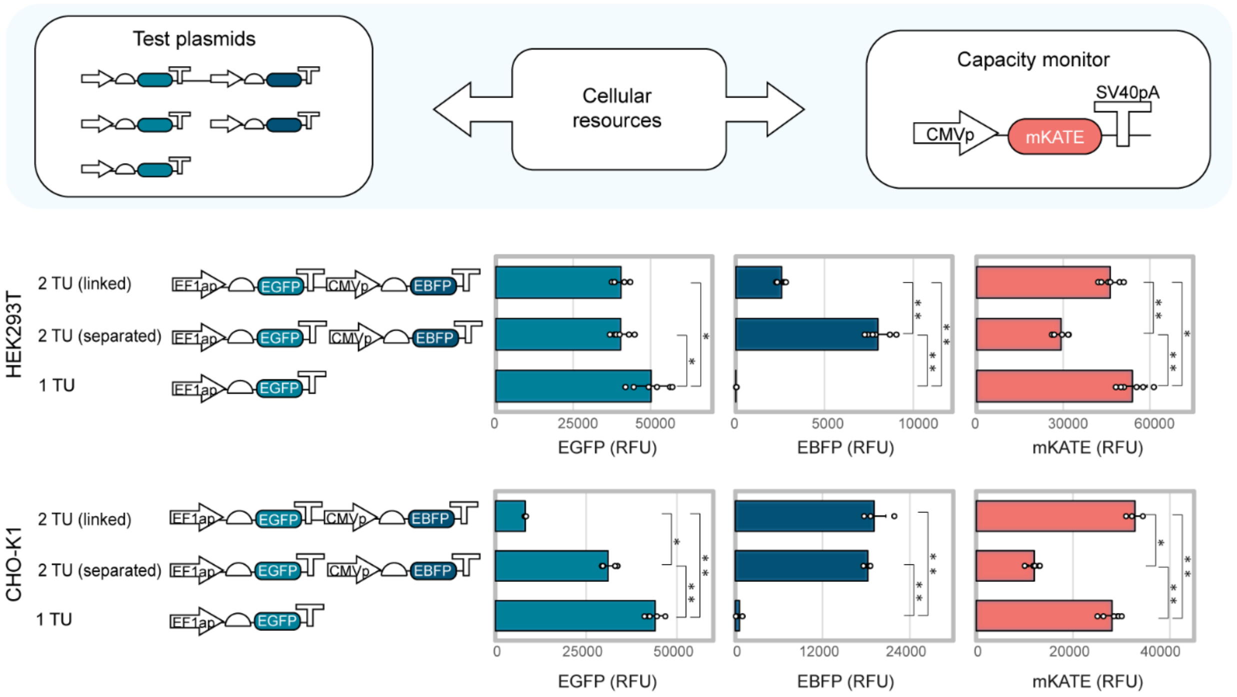

Fig. S11. Resource load and expression output of multi-gene constructs with different syntax. EGFP (left), EBFP (middle) and mKATE (right) output expressed from constructs where 1 TU, 2 TUs placed on two plasmids, or 2 TUs placed on the same plasmid are considered in HEK293T (upper panel) and CHO-K1 (bottom panel) cells. Test plasmid and capacity monitor expression levels are reported as mean RFU ± std. Number of biological repeats for each sample is reported in Supplementary data file 3. Two sided Mann-Whitney test *P* value: ****<0.0001, ***<0.0005, **<0.005, *<0.05. Exact P values can be found in Supplementary data file 4. Data were analysed as described in the Methods section and Supplementary Note 1. Source data are provided in the Source Data file.

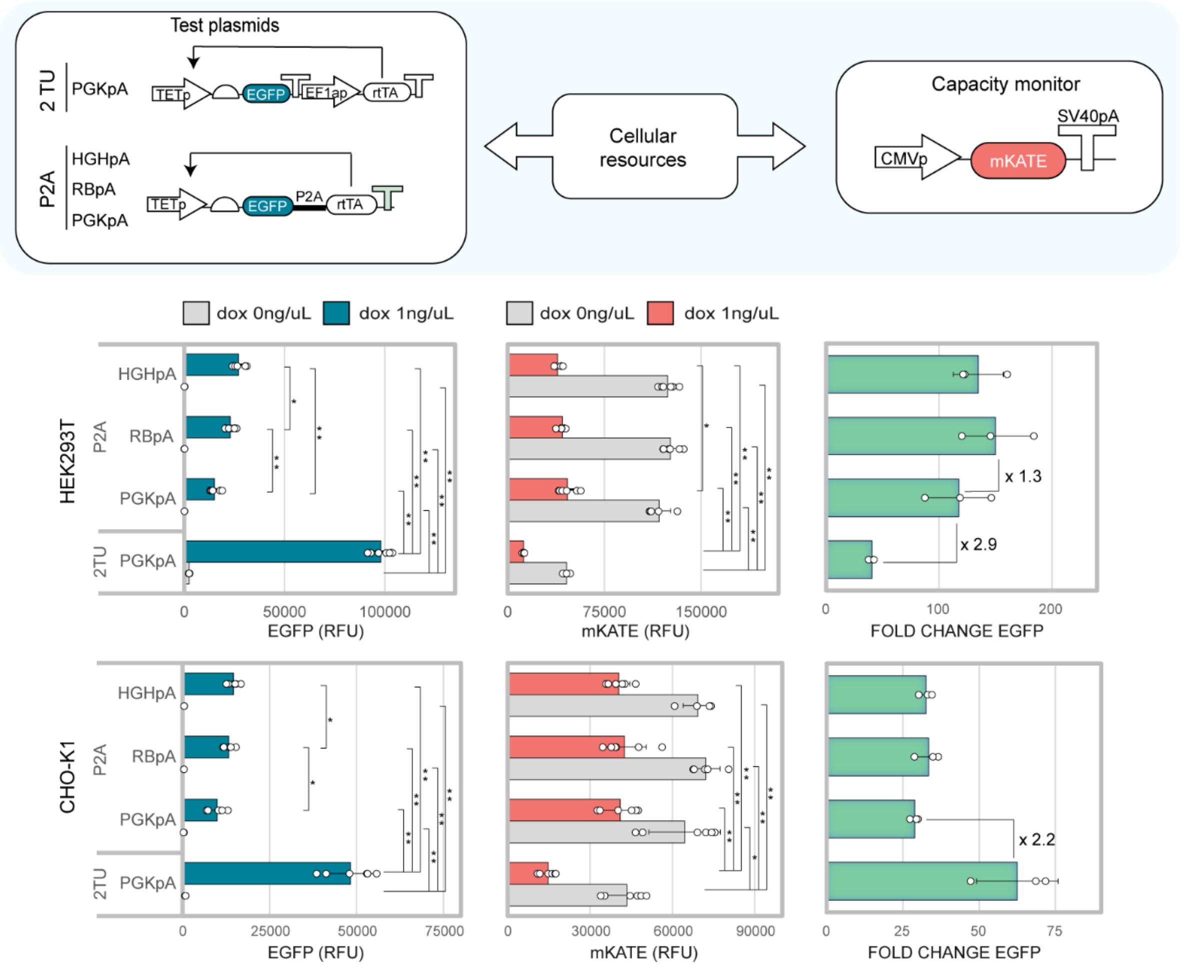

Fig. S12. Resource-aware construct design is useful to optimise genetic circuit performance. Minimisation of the resource load of the TET-ON inducible system by use of a P2A sequence. EGFP (left), mKATE (middle), and fold change – calculated as the ratio of EGFP in the presence and absence of doxycycline induction - (right) are shown. Number of biological repeats for each sample is reported in Supplementary data file 3. Two sided Mann-Whitney test *P* value: ****<0.0001, ***<0.0005, **<0.005, *<0.05. Exact P values can be found in Supplementary data file 4. Data were analysed as described in the Methods section and Supplementary Note 1. Source data are provided in the Source Data file.

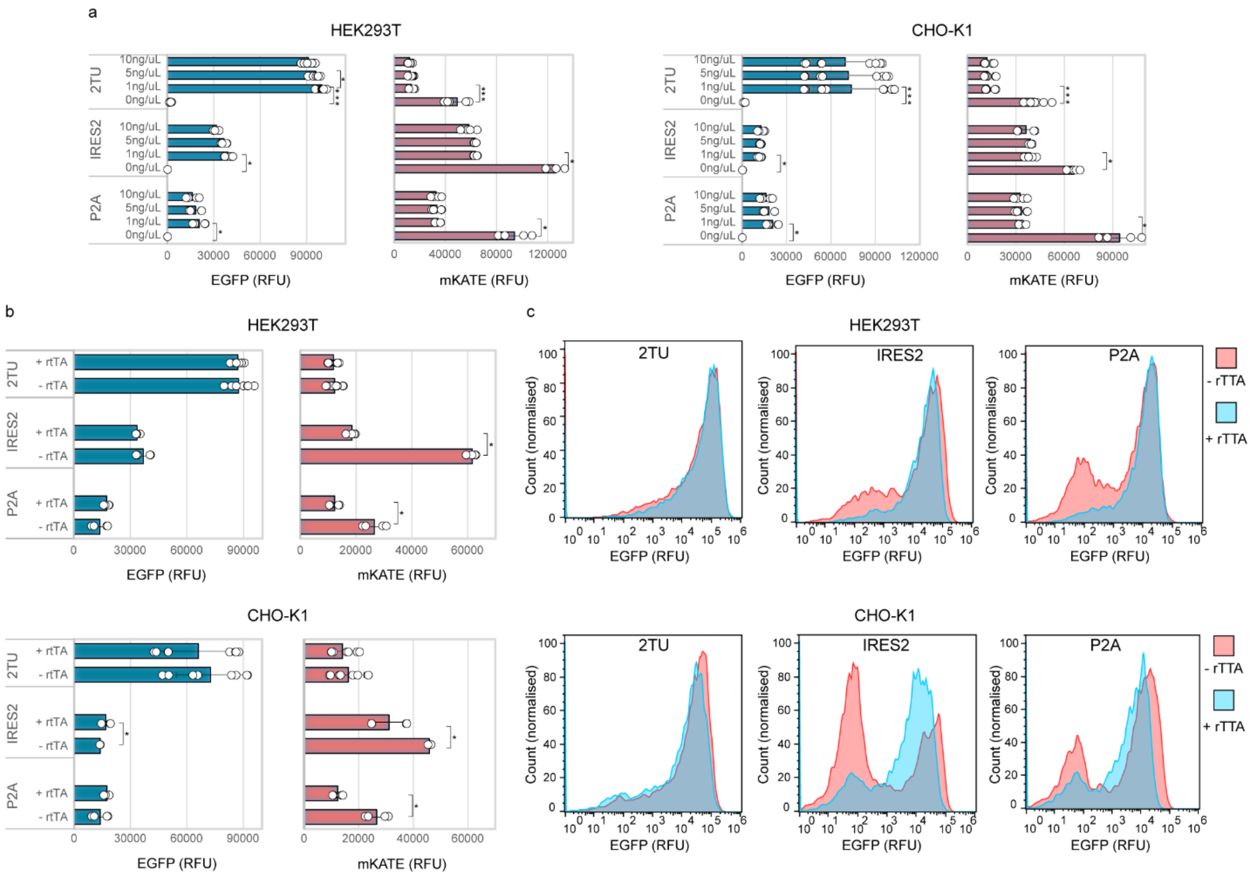

Fig. S13. Induction of three selected TET-ON designs. (a) Effect of increasing concentration of doxycycline on three selected TET-ON designs in HEK293T (left panels) and CHO-K1 (right panels). **(b)** Impact of additional rtTA transactivator on three selected TET-ON designs in HEK293T (top panels) and CHO-K1 (bottom panels). **(c)** EGFP fluorescence histograms for three selected TET-ON designs with or without co-transfection of a rtTA transactivator-expressing plasmid in HEK293T (top panels) and CHO-K1 (bottom panels). Test plasmid and capacity monitor expression levels are reported as mean RFU ± std. Number of biological repeats for each sample is reported in Supplementary data file 3. Two sided Mann-Whitney test P value: ****<0.0001, ***<0.0005, **<0.005, *<0.05. Exact P values can be found in Supplementary data file 4. Data were analysed as described in the Methods section and Supplementary Note 1. Source data are provided in the Source Data file.

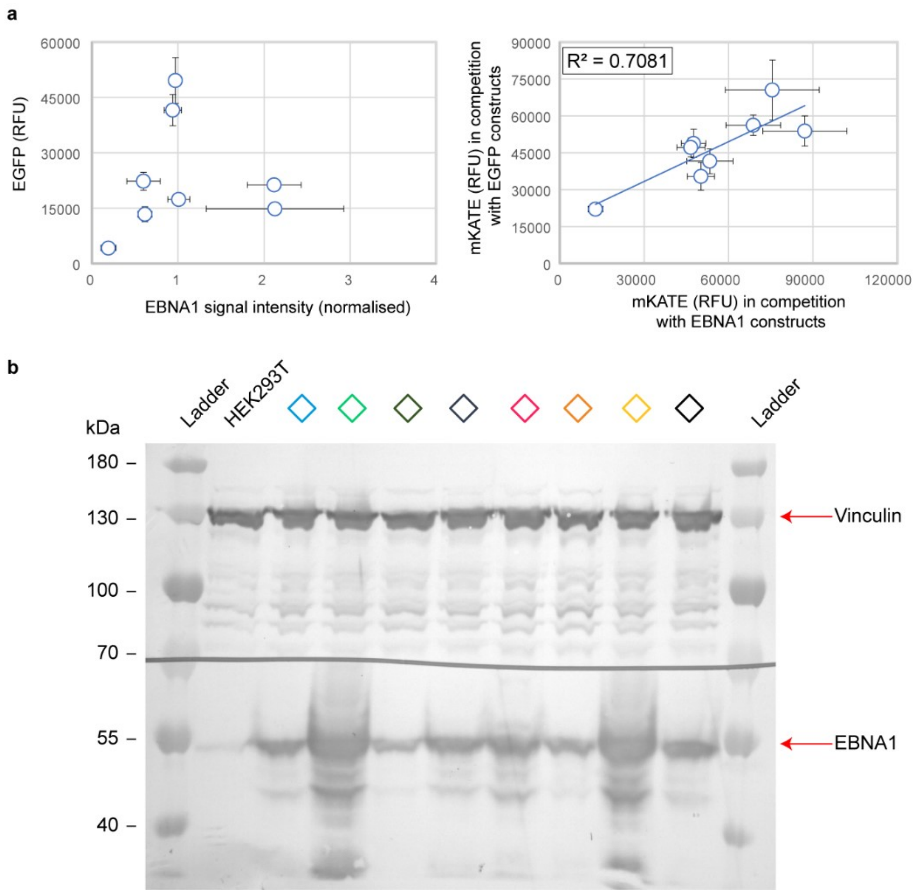

Fig. S14. Output and capacity of EBNA1 designs in HEK293T. (a) Correlation between EGFP expression (RFU) and EBNA1 expression (normalised signal intensity) for the selected designs in Fig. 4 in HEK293T (left panel). Correlation between mKATE expression in competition with EGFP (RFU) and mKATE expression in competition with EBNA1 (RFU) for the selected designs in Fig. 4b in HEK293T (right panel). **(b)** EBNA1 (test plasmid) and Vinculin (internal control) expression for the selected EBNA1-expressing designs detected by Western blot. Capacity monitor expression is reported as mean RFU ± std. EBNA1 expression is reported as normalised signal intensity and was calculated as described in the Methods section. Number of biological repeats for each sample is reported in Supplementary data file 3. Data were analysed as described in the Methods section and in Supplementary Note 1. Source data are provided in the Source Data file.

Supplementary data files.

Supplementary data file 1. Plasmid list. The file lists all the plasmids used in the research.
Supplementary data file 2. Part sequences. The file reports all the part sequences used in the research.
Supplementary data file 3. Transfection details and number of repeats. The file provides information on the experimental parameters adopted for transfection and number of repeats for each experiment.
Supplementary data file 4. Exact P values for all figures in the manuscript. The file reports the exact p values for the significance bars plotted in each figure.
Supplementary data file 5. List of primers used in this study. The file reports the sequences of the primers used in this study.

Supplementary Note 1. Flow cytometry data analysis

Single cells were manually gated and exported in FCS format after compensation using FlowJo. Further analyses were performed by custom python-code using the FlowCytometryTools library[1] and the Scipy ecosystem[2]. Fluorescence values were transformed using the Logicle transformation with default parameters[3]. The probability that a cell with value x of a marker i (EGFP, mKate or BFP) is expressing that particular marker was defined as:

$$P_i(x) = \begin{cases} 0 & if\ x \leq x_0 \\ 3\left(\frac{x - x_0}{x_1 - x_0}\right)^2 - 2\left(\frac{x - x_0}{x_1 - x_0}\right)^3 & if\ x_0 < x \leq x_1 \\ 1 & if\ x > x_1 \end{cases} \quad (1).$$

Eq. (1) implements a fuzzy clustering scheme. Cells with fluorescence values above x_1 or below x_0 are classified respectively as expressing or not-expressing the *i-th* marker with complete confidence. For cells in the region between X0 and X1, these are considered as expressing the marker with a probability that smoothly increases for increasing fluorescence values. In order to define the thresholds x_0 and x_1, cells were first classified into 2 clusters using the K-means algorithm, and then the following equations were used:

$$x_0 = x_{\rho_{min}} - \frac{\rho_{min}}{\sigma_{max}}\left(x_{\rho_{min}} - x_{\sigma_{max}}\right) \quad (2),$$

$$x_1 = x_{\rho_{min}} + \frac{\rho_{min}}{\upsilon_{max}}\left(x_{\rho_{min}} - x_{\upsilon_{max}}\right) \quad (3),$$

Where, $\rho_{min}$is the minimum of the density in the region between the centroids of the two clusters estimated by the K-means algorithm; σ_{max} and υ_{max} are the maximum of density in the same region for cells belonging respectively to the cluster with lower/higher centroid; and $x_{\rho_{min}}$, $x_{\sigma_{max}}$, and $x_{\upsilon_{max}}$ are the value of the marker corresponding to the minimum/maximum of density. According to Eq. (2)-(3), the region of uncertain classification has maximum width for flat density profile ($\rho_{min} = \sigma_{max} = \upsilon_{max}$), e.g. when the threshold separating expressing from non-expressing cells is intrinsically ill-defined. Fig. S15 shows the results of the fuzzy-clustering procedure as an illustrative example. The values of marker expressions used for further analysis were calculated as weighted averages using the weighting factor $1 - \left(1 - P_{GFP}(x)\right)\left(1 - P_{RFP}(x)\right)$. Since $\left(1 - P_i(x)\right)$ corresponds to the probability of not-expressing the i-th marker for a cell with fluorescence value equal to i, the previous formula implements an OR gate with fuzzy logic. With respect to standard analysis methods using OR-gates with manual thresholds, the procedure implemented here has two advantages. Firstly, the thresholds between cells expressing or not-expressing each marker are automatically defined on the base of Eq. (2)-(3), reducing the risk of a possible bias introduced by the operator. Secondly, in cases where a blunt

separation between expressing/not-expressing cells does not exist, the results of a hard-clustering scheme, as a manual OR-gate, depends on thresholds that are intrinsically ill-defined. The fuzzy-clustering procedure adopted here better reflects the uncertain classification of cells in these transition regions (cells in the region between x_0 and x_1 are classified as expressing the marker with a probability dictated by Eq.1).

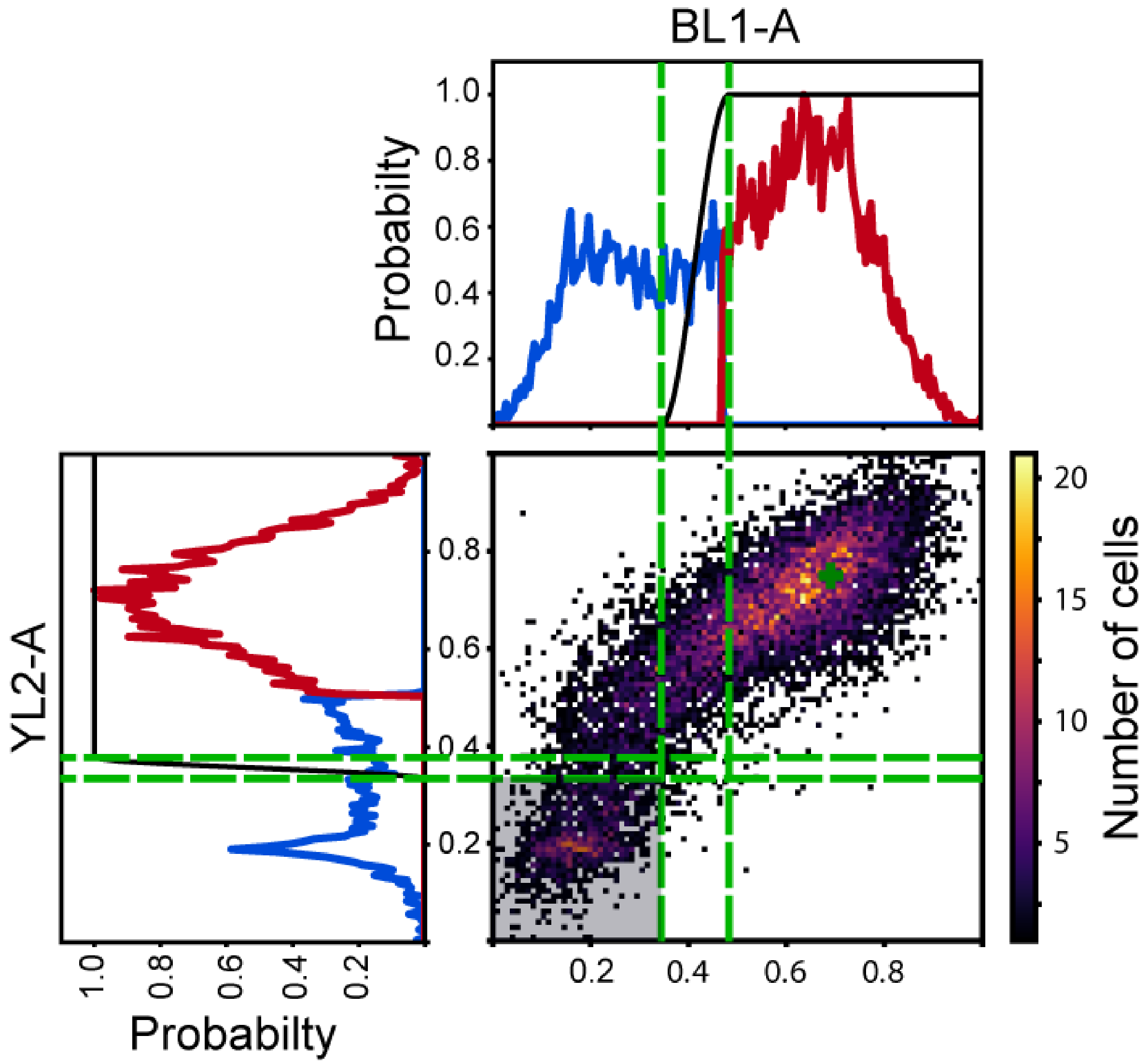

Fig. S15. Representative example of the flow cytometry data analysis. 2-dimensional and 1-dimensional density plots are shown for markers BL1-A and YL2-A as a representative example in HEK293T cells. Fluorescence values were transformed using the Logicle function. In the 1-dimensional histogram, the blue/red part of the density profile corresponds to K-means classification in 2 clusters. The black line shows the probability that a cell is considered as expressing that particular marker as defined by Eq. (1) in Supplementary note 1. The green dashed lines show the positions of the thresholds $x_{\sigma_{max}}$ and $x_{\upsilon_{max}}$, which delimit the region of uncertain classification. This region is wider for BL1-A than for YL2-A, reflecting the fact that density peaks are better separated for YL2-A than for BL1-A. The green plus sign in the 2-density plot highlights the position of the weighted average for the two markers. Cells with fluorescence values in the grey-shaded region at the bottom left of the density plot, do not contribute to this weighted average, as the probability of being in the expressing state for these cells is zero.

Supplementary Note 2. Resource competition profiles are promoter- and cell line-specific.

While testing the effect that promoter strength imposes on cellular resources, we observed interesting promoter-specific and cell line-specific effects. We adopted different capacity monitor configurations to confirm that the data obtained from the library of promoters was not capacity monitor specific (Fig. S4). When tested with the promoter library, the additional monitor designs displayed similar behaviour to the original CMV-mKATE capacity monitor, with increasing transcriptional rate from the test plasmid leading to decreased capacity monitor expression. Interestingly, upon co-transfection of the UBp-mKATE capacity monitor and the UBp-EGFP test plasmid in HEK293T, we observed an unexpectedly large decrease in the capacity monitor expression levels compared to what would be inferred from the test plasmid output. This was not observed for other pairs of competing identical promoters (i.e. CMV-mKATE vs CMV-EGFP, SV40p-mKATE vs SV40p-EGFP, EF1ap-mKATE vs EF1ap-EGFP). This may be due to UBp-specific transcription factors being limiting in HEK293T[4,5]. The impact of the genetic background is also worth noting, as this was not observed in CHO-K1, hinting that different cell lines have a different abundance of specific types of resources[6,7]. These data indicate how promoter-specific effects can add complexity to the promoter-based competition landscape and call for a thorough cell line-specific characterisation of resource composition and abundance.

Supplementary Note 3. PolyA role in shaping resource competition

Our findings indicate that the effect of polyAs on the expression of a competing cassette is cell-line dependent. To further characterise the role of polyAs in shaping resource competition, we cloned different capacity monitor bearing one of four polyAs (i.e. SV40pA_rv, HGHpA, PGKpA and RBpA). We tested the novel monitor designs in competition with a few shortlisted test designs bearing EF1ap-Kz1-EGFP and one of the five polyAs previously adopted. Our data (Fig. S6) demonstrate that the trend observed in the monitor expression is cell line- and polyA-dependent. In CHO-K1, we noticed decreasing monitor expression with increasing EGFP due to a change of polyA. In HEK293T, a similar trend was also observed for the monitors bearing PGKpA, RBpA, and HGHpA. However, for the monitors with SV40pA and SV40pA_rv, we observed the opposite behaviour, where increasing EGFP outputs led to an increase in capacity monitor expression.

PolyAs are involved in transcriptional termination, mRNA stability, and translation efficiency[8]. Our results suggest a resource bottleneck in at least one of these processes. Accounting for a resource bottleneck explains why polyAs driving a higher EGFP expression led to decreased expression of the competing capacity monitor. To explain cell-line and polyA specific effects we speculate that the availability of critical cellular factors involved in polyA-mediated biological processes might be crucial in understanding this complexity. An example of cellular factors interacting with polyAs are the cytoplasmic polyA-binding proteins (i.e. PABP), involved in mRNA stability and translation. PABPs bind the polyA, protecting the mRNAs from cellular deacetylation

complexes, which trim polyA tails, and increasing mRNA stability[8]. PAPBs also bridge the interaction between the polyA tail and eIF4G at the 5`-UTR of the mRNA, promoting mRNA circularisation and enhancing translation, something referred to as the closed-loop translation initiation model[9]. Evidence of cell line-dependent PAPB availability and polyA sequence-dependent PAPB activity has been previously reported[10,11]. Our work provides the first evidence for cell line- and sequence-dependent impact of polyAs in shaping resource competition profiles. To pinpoint which specific resources (i.e. the ones involved in transcriptional termination, mRNA stability or translation regulation) are the cause of the observed behaviour will require further experimental characterisation.

Supplementary References

1 Eugene Yurtsev, J. F., & Jeff Gore. *FlowCytometryTools: Version 0.4.5 (v0.4.5)*, 2015).

2 Virtanen, P. *et al.* SciPy 1.0: fundamental algorithms for scientific computing in Python. *Nat Methods* 17, 261-272, doi:10.1038/s41592-019-0686-2 (2020).

3 Parks, D. R., Roederer, M. & Moore, W. A. A new "Logicle" display method avoids deceptive effects of logarithmic scaling for low signals and compensated data. *Cytometry A* 69, 541-551, doi:10.1002/cyto.a.20258 (2006).

4 Parvin, J. D., Timmers, H. T. & Sharp, P. A. Promoter specificity of basal transcription factors. *Cell* 68, 1135-1144, doi:10.1016/0092-8674(92)90084-p (1992).

5 Hollenhorst, P. C., Jones, D. A. & Graves, B. J. Expression profiles frame the promoter specificity dilemma of the ETS family of transcription factors. *Nucleic Acids Res* 32, 5693-5702, doi:10.1093/nar/gkh906 (2004).

6 Jones, R. D. *et al.* An endoribonuclease-based feedforward controller for decoupling resource-limited genetic modules in mammalian cells. *Nat Commun* 11, 5690, doi:10.1038/s41467-020-19126-9 (2020).

7 Cardinale, S., Joachimiak, M. P. & Arkin, A. P. Effects of genetic variation on the E. coli host-circuit interface. *Cell Rep* 4, 231-237, doi:10.1016/j.celrep.2013.06.023 (2013)

8 Passmore, L. A. & Coller, J. Roles of mRNA poly(A) tails in regulation of eukaryotic gene expression. *Nat Rev Mol Cell Biol*, doi:10.1038/s41580-021-00417-y (2021)

9 Wang, Z. & Kiledjian, M. The poly(A)-binding protein and an mRNA stability protein jointly regulate an endoribonuclease activity. *Mol Cell Biol* 20, 6334-6341, doi:10.1128/MCB.20.17.6334-6341.2000 (2000)

10 Adivarahan, S. *et al.* Spatial Organization of Single mRNPs at Different Stages of the Gene Expression Pathway. *Mol Cell* 72, 727-738 e725, doi:10.1016/j.molcel.2018.10.010 (2018).

11 Castellano, L. A. & Bazzini, A. A. Poly(A) tails: longer is not always better. *Nat Struct Mol Biol* 24, 1010-1011, doi:10.1038/nsmb.3509 (2017).
