# Supplementary material for: Resource-aware construct design in mammalian cells
Source: Nat Commun. 2023 Jun 16;14:3576. doi: 10.1038/s41467-023-39252-4 (PMC10275982; doi:10.1038/s41467-023-39252-4)
Supplement: Supplementary file 2 — Description of Additional Supplementary Files [file 41467_2023_39252_MOESM2_ESM.pdf]

**Title: Supplementary data 1.**
**Description: Plasmid list.** The file lists all the plasmids used in the research.

**Title: Supplementary data 2.**
**Description: Part sequences.** The file reports all the part sequences used in the research.

**Title: Supplementary data 3.**
**Description: Transfection details and number of repeats.** The file provides information on the experimental parameters adopted for transfection and number of repeats for each experiment.

**Title: Supplementary data 4.**
**Description: Exact P values for all figures in the manuscript.** The file reports the exact p values for the significance bars plotted in each figure.

**Title: Supplementary data 5.**
**Description: List of primers used in this study.** The file reports the sequences of the primers used in this study.
