# Supplementary material for: Resource-aware construct design in mammalian cells
Source: Nat Commun. 2023 Jun 16;14:3576. doi: 10.1038/s41467-023-39252-4 (PMC10275982; doi:10.1038/s41467-023-39252-4)
Supplement: Supplementary file 3 — Reporting Summary [file 41467_2023_39252_MOESM3_ESM.pdf]

nature portfolio

Corresponding author(s): Ceroni Francesca

Last updated by author(s): 22-05-2023

Reporting Summary

Nature Portfolio wishes to improve the reproducibility of the work that we publish. This form provides structure for consistency and transparency in reporting. For further information on Nature Portfolio policies, see our Editorial Policies and the Editorial Policy Checklist.

Statistics

For all statistical analyses, confirm that the following items are present in the figure legend, table legend, main text, or Methods section.

n/a	Confirmed	
☐	☒	The exact sample size (n) for each experimental group/condition, given as a discrete number and unit of measurement
☐	☒	A statement on whether measurements were taken from distinct samples or whether the same sample was measured repeatedly
☐	☒	The statistical test(s) used AND whether they are one- or two-sided<br>*Only common tests should be described solely by name; describe more complex techniques in the Methods section.*
☐	☒	A description of all covariates tested
☐	☒	A description of any assumptions or corrections, such as tests of normality and adjustment for multiple comparisons
☐	☒	A full description of the statistical parameters including central tendency (e.g. means) or other basic estimates (e.g. regression coefficient) AND variation (e.g. standard deviation) or associated estimates of uncertainty (e.g. confidence intervals)
☐	☒	For null hypothesis testing, the test statistic (e.g. F, t, r) with confidence intervals, effect sizes, degrees of freedom and P value noted<br>*Give P values as exact values whenever suitable.*
☐	☒	For Bayesian analysis, information on the choice of priors and Markov chain Monte Carlo settings
☐	☒	For hierarchical and complex designs, identification of the appropriate level for tests and full reporting of outcomes
☒	☐	Estimates of effect sizes (e.g. Cohen's d, Pearson's r), indicating how they were calculated

Our web collection on statistics for biologists contains articles on many of the points above.

Software and code

Policy information about availability of computer code

Data collection	Attune NxT flow cytometer (ThermoFisher). Single cells were manually gated and exported in FCS format after compensation using FlowJo (version number: Flowjo v10.6.2) (full description is available in the methods section of the manuscript). Western data were analysed using ImageJ (reference provided in the methods section of the manuscript). Flow data analysis was performed using the code available at https://github.com/sfurini/gating_ResourceAwareDesign.git and doi: doi.org/10.5281/zenodo.7956982.
Data analysis	Fluorescent data were manually gated and exported in FCS format after compensation using Flow Jo (version number: Flowjo v10.6.2). Further analysis was performed using custom Python code (available at https://github.com/sfurini/gating_ResourceAwareDesign.git and doi: doi.org/10.5281/zenodo.7956982.) using FlowCytometry tools and Scipy ecosystem. Fluorescence values were transformed using the Logicle transformation with default parameters. Full description is available in Supplementary note 1.

For manuscripts utilizing custom algorithms or software that are central to the research but not yet described in published literature, software must be made available to editors and reviewers. We strongly encourage code deposition in a community repository (e.g. GitHub). See the Nature Portfolio guidelines for submitting code & software for further information.

Data

Policy information about availability of data

All manuscripts must include a data availability statement. This statement should provide the following information, where applicable:

- Accession codes, unique identifiers, or web links for publicly available datasets
- A description of any restrictions on data availability
- For clinical datasets or third party data, please ensure that the statement adheres to our policy

Data availability statement. All source data for the data sets and figures presented in the manuscript are available at "Roberto, Di Blasi; Mara, Pisani; Tedeschi, Fabiana; Marbiah, Masue; Polizzi, Karen Marie; Furini, Simone; et al. (2023): Resource-aware construct design in mammalian cells. Figshare. Dataset. https://doi.org/10.6084/m9.figshare.22659211". Constructs and plasmid maps are available upon request to the corresponding author.

Research involving human participants, their data, or biological material

Policy information about studies with human participants or human data. See also policy information about sex, gender (identity/presentation), and sexual orientation and race, ethnicity and racism.

Reporting on sex and gender	N/A
Reporting on race, ethnicity, or other socially relevant groupings	N/A
Population characteristics	N/A
Recruitment	N/A
Ethics oversight	N/A

Note that full information on the approval of the study protocol must also be provided in the manuscript.

Field-specific reporting

Please select the one below that is the best fit for your research. If you are not sure, read the appropriate sections before making your selection.

☒ Life sciences ☐ Behavioural & social sciences ☐ Ecological, evolutionary & environmental sciences

For a reference copy of the document with all sections, see nature.com/documents/nr-reporting-summary-flat.pdf

Life sciences study design

All studies must disclose on these points even when the disclosure is negative.

Sample size	No sample size calculation was performed. Number of repeats and days of performed experiments are reported in Supplementary Table 3.
Data exclusions	All data points lower than Q1-1.5*IQR and higher than Q3-1.5*IQR-where IQR stand for interquartile range-where considered outliers and excluded from further analysis.
Replication	Samples have different numbers of replications (reported in Supplementary Table 3). All samples were repeated on different days (biological independent repeats). All replications were successful but some experiments yielded outliers. All data points lower than Q1-1.5*IQR and higher than Q3-1.5*IQR-where IQR stand for interquartile range-where considered outliers and excluded from further analysis.
Randomization	N/A<br>Samples were grouped based on experimental design where needed and tested all together where needed. No specific randomization was required for our study.
Blinding	N/A<br>No blinding was required for our study.

Reporting for specific materials, systems and methods

We require information from authors about some types of materials, experimental systems and methods used in many studies. Here, indicate whether each material, system or method listed is relevant to your study. If you are not sure if a list item applies to your research, read the appropriate section before selecting a response.

Materials & experimental systems

n/a	Involved in the study
☐	☒ Antibodies
☐	☒ Eukaryotic cell lines
☒	☐ Palaeontology and archaeology
☒	☐ Animals and other organisms
☒	☐ Clinical data
☒	☐ Dual use research of concern
☒	☐ Plants

Methods

n/a	Involved in the study
☒	☐ ChIP-seq
☐	☒ Flow cytometry
☒	☐ MRI-based neuroimaging

Antibodies

Antibodies used	Proteins were probed for anti-EBNA1 (Merck, clone 1H4, monoclonal, cat MABF2800, dilution 1:1000) and a loading control, anti-Vinculin (Sigma, clone hVIN-1, monoclonal, cat V9264, dilution 1:5000). Secondary antibodies (Life Technologies, Goat anti-Rat IgG A18868, polyclonal, and Abcam Rabbit Anti-Mouse IgG H&L (Alkaline Phosphatase) ab6729, dilutions 1:10000) were detected using the Novex™ AP Chromogenic Substrate (Invitrogen).
Validation	Western blot validation for anti-EBNA1 antibody is described in Grasser et al (1994). Blood. 84 (11):3792-8. "no signal was obtained with cell extracts derived from EBNA2A-expressing cells or cells infected with the wild type baculovirus".<br>Manufacturer`s state: "Anti-vinculin antibody, mouse monoclonal has been used in immunoblotting" and "specifically labels vinculin at cell-cell and cell-substrate contact. Reacts strongly with human vinculin. Shows cross-reactivity with smooth mussle metavinculin".

Eukaryotic cell lines

Policy information about cell lines and Sex and Gender in Research

Cell line source(s)	HEK293T and CHO-K1 ATCC
Authentication	Cells were purchased from ATCC and not authenticated afterwards.
Mycoplasma contamination	HEK293T tested negative to mycoplasma. CHO-K1 were not mycoplasma tested.
Commonly misidentified lines<br>(See ICLAC register)	N/A

Flow Cytometry

Plots

Confirm that:

☒ The axis labels state the marker and fluorochrome used (e.g. CD4-FITC).

☒ The axis scales are clearly visible. Include numbers along axes only for bottom left plot of group (a 'group' is an analysis of identical markers).

☒ All plots are contour plots with outliers or pseudocolor plots.

☒ A numerical value for number of cells or percentage (with statistics) is provided.

Methodology

Sample preparation	HEK293T and CHO-K1 cells were detached, centrifuged, resuspended in DPBS (GIBCO) and filtered to disrupt any cell clumps.
Instrument	Attune NxT flow cytometer (Thermo Fisher Scientific)
Software	Fluorescent data were manually gated and exported in FCS format after compensation using Flow Jo (version number: Flowjo v10.6.2). Further analysis was performed using custom Python code (available at https://github.com/sfurini/gating_ResourceAwareDesign.git) using FlowCytometry tools and Scipy ecosystem. Fluorescence values were transformed using the Logicle transformation with default parameters. Full description is available in Supplementary note 1.
Cell population abundance	Cell population abundance depended on the transfected sample. For HEK293T it was around 85%-95%, for CHO-K1 between 60 and 80%.
Gating strategy	Transfected cells were gated using an OR gate approach with fuzzy logic (see Supplementary Note 1). Code used for analysis and gating is available at https://github.com/sfurini/gating_ResourceAwareDesign.git

☒ Tick this box to confirm that a figure exemplifying the gating strategy is provided in the Supplementary Information.
